# Effects of Perioperative Epidural Analgesia on Cancer Recurrence and Survival

**DOI:** 10.3389/fonc.2021.798435

**Published:** 2022-01-05

**Authors:** Donghang Zhang, Jingyao Jiang, Jin Liu, Tao Zhu, Han Huang, Cheng Zhou

**Affiliations:** ^1^ Department of Anesthesiology, West China Hospital of Sichuan University, Chengdu, China; ^2^ Laboratory of Anesthesia and Critical Care Medicine, National-Local Joint Engineering Research Centre of Translational Medicine of Anesthesiology, West China Hospital of Sichuan University, Chengdu, China; ^3^ Department of Anesthesiology & Key Laboratory of Birth Defects and Related Diseases of Women and Children, Ministry of Education, West China Second Hospital of Sichuan University, Chengdu, China

**Keywords:** epidural analgesia, cancer recurrence, cancer survival, cancer surgeries, oncological outcomes

## Abstract

Surgical resection is the main curative avenue for various cancers. Unfortunately, cancer recurrence following surgery is commonly seen, and typically results in refractory disease and death. Currently, there is no consensus whether perioperative epidural analgesia (EA), including intraoperative and postoperative epidural analgesia, is beneficial or harmful on cancer recurrence and survival. Although controversial, mounting evidence from both clinical and animal studies have reported perioperative EA can improve cancer recurrence and survival *via* many aspects, including modulating the immune/inflammation response and reducing the use of anesthetic agents like inhalation anesthetics and opioids, which are independent risk factors for cancer recurrence. However, these results depend on the cancer types, cancer staging, patients age, opioids use, and the duration of follow-up. This review will summarize the effects of perioperative EA on the oncological outcomes of patients after cancer surgery.

## Introduction

Cancer has become a major cause of death worldwide, while metastasis and/or recurrence is the major cause of death from cancer (1–3). Surgical resection of primary solid tumors remains a cornerstone of cancer treatment (4). However, the surgical process is associated with immunosuppression, which may generate a high vulnerability for tumor worse progression (4–6). Meanwhile, several drugs, such as volatile anesthetics and opioids during perioperative periods were also suggested to be implicated in immunosuppression and cancer recurrence (7). Regional anesthesia (RA), such as epidural anesthesia, spinal aesthesia, paravertebral block, can provide effective pain relief preoperatively (8). The adjunctive use of RA for general anesthesia is believed to decrease the requirement of opioids and general anesthetics consumption, and attenuate surgical-related stress and immunosuppression (9). Therefore, RA is theoretically suggested to have potential impacts on oncological outcomes in patients underwent cancer surgeries. Among various regional techniques in RA, the most commonly used for cancer surgery is perioperative epidural analgesia (EA), including both intraoperative or postoperative use. Currently, there is no definitive consensus whether perioperative EA is beneficial to cancer recurrence and survival. Cancer types and staging may be the major contributors to these inconsistent results. In this review, we summarized the current evidences regarding the effects of perioperative EA on recurrence and survival for various cancer types. Study characteristics were summarized in **Table 1**.

**Table 1:** Study characteristics.

Study	Study design	Number of participants	Age (years)*	Cancer type	Surgery approach	Analgesia period and LAs used	Follow-up duration	Oncological outcomes	Association between EA and outcomes
Wu 2020 (10)	Retrospective	2,748	EA group: 69 ± 14; Non-EA group: 68 ± 13	Colon cancer (stage I-III)	NA	Intraoperative and postoperative analgesia (0.25% or 0.5% bupivacaine)	46.1 months	Recurrence-free survival and overall survival	No
Wu 2021 (11)	Retrospective	1,282	EA group: 69 ± 13; Non-EA group: 66 ± 13	Rectal cancer (stage I-III)	NA	Intraoperative and postoperative analgesia (0.25% or 0.5% bupivacaine)	46.1 months	Cancer recurrence, all−cause mortality and cancer−specific mortality	No
Tai 2018 (12)	Retrospective	999	EA group: 65 ± 13; Non-EA group: 66 ± 14	Colorectal cancer (stage IV)	NA	Intraoperative and postoperative analgesia (0.25% or 0.5% bupivacaine)	17.5 months	Progression-free survival and overall survival	No
Day 2012 (13)	Retrospective	280	EA group: 72; Non-EA group: 70	Colorectal cancer	Laparoscopic	Intraoperative and postoperative analgesia (0.15% bupivacaine)	37 months	Overall survival and disease-free survival	No
Gupta 2011 (14)	Retrospective	655	EA group: 71.4 (21-96); Non-EA group: 73.2 (38-92)	Colorectal cancer (stage I-III)	Open	Intraoperative and postoperative analgesia	2.68 years	All-cause mortality	Yes for all-cause mortality of rectal but No for colon cancer
Gottschalk 2010 (15)	Retrospective	669	EA group: 65 (54-74); Non-EA group: 63 (55-72)	Colorectal cancer	NA	Intraoperative and postoperative analgesia	1.8 years	Cancer recurrence	No
Falk 2021 (16)	Prospective	221	EA group: 67.9 (41-80); Non-EA group: 67.2 (39-81)	Colorectal cancer	NA	Intraoperative and postoperative analgesia	5 years	Disease-free survival	No
Vogelaar 2015 (17)	Retrospective	588	EA group: 70 ± 12; Non-EA group: 71 ± 13	Colon cancer (stage I-IV)	NA	Intraoperative and postoperative analgesia	53 months	Survival	Yes
Holler 2013 (18)	Retrospective	749	NA	Colorectal cancer	NA	Intraoperative and postoperative analgesia	5 years	Survival	Yes
Christopherson 2008 (19)	Prospective	177	EA group: 68.6 ± 7.7; Non-EA group: 69.1 ± 7.8	Colon cancer	NA	Intraoperative and postoperative analgesia (0.5% bupivacaine)	10 years	Survival	Yes for patients without metastases before 1.46 years
Cummings 2012 (20)	Retrospective	42,151	EA group: 77.1 (72.2-82.1); Non-EA group: 78.1 (72.8-83.6)	Nonmetastatic colorectal cancer	Open	Intraoperative and postoperative analgesia	4 years	Cancer recurrence and survival	Yes for survival; No for cancer recurrence
Cummings 2014 (21)	Retrospective	2,745	EA group: 76.5 (72.0-81.8); Non-EA group: 76.9 (72.5-82.3)	Nonmetastatic gastric cancer	Open	Intraoperative and postoperative analgesia	4 years	Cancer recurrence and survival	No
Shin 2017 (22)	Retrospective	3,799	EA group: 57.5 ± 11.7; Non-EA group: 59.6 ± 11.6	Gastric cancer	NA	Intraoperative and postoperative analgesia (0.15% ropivacaine)	53.3 months	Cancer recurrence and mortality	No
Wang 2016 (23)	Retrospective	273	EA group: 67 (59-76); Non-EA group: 70 (63-78)	Gastric cancer	NA	Intraoperative and postoperative analgesia (0.25% levobupivacaine or ropivacaine)	8 years	Survival	Yes for patients < 64 years
Pei 2020 (24)	Retrospective	194	< 70	Gastric cancer	NA	Intraoperative and postoperative analgesia	5 years	Overall survival	Yes
Cummings 2019 (25)	Retrospective	1,921	EA group: 73.4 ± 4.9; Non-EA group: 74.0 ± 5.2	Esophageal cancer	NA	Intraoperative and postoperative analgesia	2.2 years	Survival	Yes
Heinrich 2015 (26)	Retrospective	153	EA group: 61 (53-69); Non-EA group: 65 (57.5-72.5)	Esophageal cancer	NA	Postoperative analgesia (0.2% ropivacaine)	5 years	Cancer recurrence and survival	No
Li 2016 (27)	Retrospective	356	NA	Esophageal cancer	NA	Postoperative analgesia (0.125% ropivacaine)	34.9 months	Cancer recurrence and overall survival	No
Hiller 2014 (28)	Retrospective	140	EA group: 67 ± 10; Non-EA group: 66 ± 11	Gastro-oesophageal cancer	NA	Intraoperative and postoperative analgesia (0.125% bupivacaine)	2 years	Cancer recurrence and survival	Yes
Xu 2021 (29)	Prospective	400	EA group: 60 ± 10; Non-EA group: 61 ± 10	Lung cancer	NA	Intraoperative (0.375% ropivacaine) and postoperative (0.12% ropivacaine) analgesia	32 months	Recurrence-free survival, overall survival and cancer-specific survival	No
Wu 2019 (30)	Retrospective	744	EA group: 64 ± 12; Non-EA group: 64 ± 11	Non-small-cell lung cancer (stage I–III)	NA	Intraoperative and postoperative analgesia (0.25% or 0.5% bupivacaine)	40.3 months	Recurrence-free and overall survival	No
de Oliveira 2011 (31)	Retrospective	182	EA group: 55 ± 12; Non-EA group: 57 ± 12	Ovarian cancer	NA	Intraoperative and postoperative analgesia or postoperative analgesia only	42 months	Cancer recurrence	Yes for intraoperative and postoperative analgesia; No for postoperative analgesia only
Elias 2015 (32)	Retrospective	194	61.0 (54.0-67.0)	Epithelial ovarian cancer (Stage III)	NA	Intraoperative and postoperative analgesia (bupivacaine)	46 months	Disease-free survival	Yes for more than 48 h of EA use
Tseng 2018 (33)	Retrospective	648	EA group: 62 (19-88); Non-EA group: 61 (30-86)	Ovarian Cancer (stage IIIB-IV)	NA	Intraoperative and postoperative analgesia or postoperative analgesia only (0.05% bupivacaine)	7 years.	Progression-free survival and overall survival	Yes
Lacassie 2013 (34)	Retrospective	80	EA group: 59 (48-65); Non-EA group: 60 (50-69)	Ovarian cancer (stage IIIC-IV)	NA	Intraoperative and postoperative analgesia (0.1%-0.5% bupivacaine)	4.9 years	Cancer recurrence and overall survival	No
Capmas 2012 (35)	Retrospective	94	EA group: 50 ± 12; Non-EA group: 56 ± 9	Ovarian cancer (advance stage)	NA	Postoperative analgesia (0.2% ropivacaine)	50 months	Recurrence-free survival and overall survival	No
Wuethrich 2010 (36)	Retrospective	261	EA group: 63 (57-67); Non-EA group: 64 (59-68)	Prostate cancer	NA	Intraoperative (0.25% bupivacaine) and postoperative (0.1% bupivacaine) analgesia	11.9 years	Biochemical recurrence-free survival, clinical progression-free survival, cancer-specific survival, and overall survival	Yes for clinical progression-free survival; No for biochemical recurrence-free survival, cancer-specific survival, or overall survival.
Wuethrich 2013 (37)	Retrospective	148	EA group: 63.61 (57.61-68.17); Non-EA group: 63.83 (59.12-67.48)	Prostate cancer	NA	Intraoperative (0.25% bupivacaine) and postoperative (0.1% bupivacaine) analgesia	14 years	Biochemical recurrence-free, local and distant recurrence-free, cancer-specific, and overall survival	No
Forget 2011 (38)	Retrospective	111	65 ± 7	Prostate cancer	NA	Intraoperative and postoperative analgesia (bupivacaine)	38 months	Biochemical recurrence-free survival	No
Tsui 2010 (39)	Prospective	99	EA group: 63.0 ± 5.5; Non-EA group: 63.9 ± 6.1	Prostate cancer	NA	Intraoperative analgesia (0.2% ropivacaine)	4.5 years	Disease-free survival	No
Biki 2008 (40)	Retrospective	225	EA group: 63 ± 5; Non-EA group: 62 ± 6	Prostate cancer	Open	Postoperative analgesia	12.8 years	Cancer recurrence	Yes
Chipollini 2018 (41)	Retrospective	430	EA group: 69; Non-EA group: 70	Bladder cancer	NA	Intraoperative and postoperative analgesia (50 mcg sufentanil)	41.4 months	Recurrence-free and cancer-specific survival	Worse outcomes
Christopher Doiron 2016 (42)	Retrospective	1,628	NA	Bladder cancer	NA	NA	5 years	Cancer-specific survival and overall survival	No
Chang 2019 (43)	Retrospective	554	EA group: 61 ± 14; Non-EA group: 61 ± 12	Hepatocellular carcinoma	NA	Intraoperative (0.25% or 0.5% bupivacaine) and postoperative (0.25% or 0.5% bupivacaine) analgesia	64.5 months	Recurrence-free and overall survival	No
Cao 2014 (44)	Retrospective	819	EA group: 48.0 ± 11.6; Non-EA group: 49.5 ± 12.1	Hepatocellular carcinoma.	NA	Postoperative analgesia (0.15% ropivacaine combined with 0.07 mg/kg per day morphine)	4.2 years	Recurrence-free survival and long-term survival	EA increased cancer recurrence but had no effect on recurrence-free survival
Gao 2019a (45)	Retrospective	225	EA group: 54 (47-60); Non-EA group: 54 (48-63)	Colorectal carcinoma liver metastases	NA	Intraoperative analgesia (0.2% ropivacaine)	5 years	Cancer recurrence	Yes
Zimmitti 2016 (46)	Retrospective	510	EA group: 58 (23-87); Non-EA group: 57 (28-86)	Colorectal carcinoma liver metastases	NA	Intraoperative and postoperative analgesia (0.075% bupivacaine)	84 months	Recurrence-free and overall survival	Yes for recurrence-free survival; No for overall survival
Call 2015 (47)	Retrospective	111	NA	Pancreatic adenocarcinoma	NA	NA	437 days	Survival	Yes
Alexander 2021 (48)	Retrospective	98	65 (41-85)	Pancreatic adenocarcinoma	NA	Intraoperative (0.375% ropivacaine) and postoperative (0.2% ropivacaine) analgesia	17.26 months	Cancer recurrence or overall survival	No

*Data are present as mean with standard deviation or median with interquartile range; EA, Epidural analgesia; NA., Not applicable.

## Effects of Surgery on Cancer Recurrence

Surgical resection is the main curative avenue for various solid cancers (1). Unfortunately, minimal residual disease may be present persistently after treatment, which can cause metastasis and recurrence (49). Meanwhile, the operation and general anesthesia process themself may facilitate the tumor metastasis and recurrence through several ways, such as stress and immune/inflammation responses, and postoperative pain (50–55). Furthermore, tissue damage caused by surgery, especially the local pro-inflammatory and wound-healing responses, were associated with local and distant recurrence (56). Additionally, postoperative pain is suggested as an important contributor to suppress immunity function, thus promoting cancer progression (49, 57). For general anesthesia, inhaled anesthetics and opioids were reported to be related to worse oncological outcomes for cancer surgeries (20). Therefore, the perioperative period represents as a critical timeframe for metastatic progression and cancer recurrence.

## Effects of Perioperative EA on Cancer Recurrence

Mounting evidence from both clinical and animal researches indicated that perioperative EA could improve cancer recurrence and survival (17, 28, 31). The underlying mechanism remains elusive, which was mainly attributed to improve immunosuppression *via* attenuating surgical stress and postoperative pain, reducing requirements for opioid and anesthetics, and direct anti-metastasis effects of local anesthetics (58).

### Perioperative EA Attenuates Surgical Stress and Pain

During and/or after the surgical resection of tumor, stress responses and pain are commonly existed and interacted, which may cause immunosuppression, thus promoting cancer recurrence (59, 60). Perioperative EA was reported to attenuate the immunosuppression by inhibiting the stress responses and/or alleviating the perioperative pain (58). Meanwhile, perioperative EA can improve the function of immunity *via* preserve and/or increase the numbers of immune cells and reduce the plasma concentrations of immune suppressive soluble factors (61–65).

### Perioperative EA Reduces Opioid and Anesthetics Requirements

Opioids were suggested to be an important factor that suppress the immune function (66, 67). For example, morphine and remifentanil suppress NK cell activity and T cell differentiation, and promote lymphocyte apoptosis (68–71). Likewise, fentanyl and sufentanil decrease NK cell activity or leukocyte migration (72–74). In addition to opioids, previous studies reported that volatile anesthetics are also independent risk factors of cancer recurrence (75–78). It is well known that perioperative EA significantly reduced the requirements for perioperative opioids and volatile anesthetics use during the cancer surgery, thus influencing the oncological outcomes (58, 79, 80).

### Direct Anti-Metastasis Effects of Local Anesthetics

Metastasis is an important factor for cancer recurrence and is the major cause of death from most malignant cancers. During the process of metastasis, tumor cells undergo several steps known as the metastatic cascade. At the primary site, tumor cells escape from the antitumor immune response, invade the surrounding parenchyma and intravasate into blood and/or lymphatic vessels, which allows them to circulate and spread. At the metastatic site, these circulating tumor cells extravasate from the blood and/or lymphatic vessel, survive and proliferate to form the metastatic tumor (3). Local anesthetics used in RA were suggested to directly inhibit the metastasis process (81, 82). For example, lidocaine has anti-growth and anti-metastatic properties towards lung cancer cells (83). Ropivacaine is demonstrated to reduce the proliferation of breast cancer cells and induce the apoptosis processes (84). Although there is no consensus whether different local anesthetics have different effects on the cancer outcomes *in vivo*, it is suggested that all local anesthetics at high concentrations are toxic to cancer cells *in vitro* with different potencies (bupivacaine > lidocaine > ropivacaine) (85). The underlying mechanism remains elusive, which may involves ion channels (86–89), inflammatory pathways (90, 91), and cancer stem cells (92, 93).

## Effects of Perioperative EA on Certain Cancer Types

In clinic, perioperative EA is commonly used for thoracic and abdominal surgeries due to many advantage aspects, such as postoperative pain management, reducing requirements for anesthetics as well as postoperative complications (58, 94, 95). However, the potential benefit of perioperative EA on cancer recurrence and survival is debated in patients undergoing thoracic and abdominal surgeries, which is suggested most likely related to cancer types. Du et al. evaluated the effects of perioperative EA on the long-term oncological outcomes in elderly patients (60 to 90 years old) with major thoracic and abdominal surgeries. The results found that, compared with general anesthesia alone with postoperative intravenous analgesia, combined epidural-general anesthesia with postoperative epidural analgesia did not improve overall or cancer-specific mortality, or the recurrence-free survival after a median follow-up duration of 66 months (96). Similarly, another retrospective study also did not support an association between postoperative EA use and recurrence-free and overall survival after abdominal cancer surgery (97). One randomized controlled trial (RCT) involving 503 patients also found that postoperative EA use had no effects on cancer-free survival after abdominal cancer surgery (98). The effects of perioperative EA on long-term oncological outcomes were varied for specific cancer surgery.

### Effects of Perioperative EA on Colorectal Cancer Surgery

A large number of retrospective studies have evaluated the effects of perioperative EA on oncological outcomes in patients underwent colorectal cancer surgeries, but the results were inconsistent. One study compared the effects of perioperative EA (combined intraoperative and postoperative analgesia) with intravenous opioid analgesia in patients receiving surgery for colon cancer (stage I to III). No association was found between perioperative EA use and cancer recurrence or death with 46.1 months duration of follow-up. However, higher level of preoperative carcinoembryonic antigen, perioperative blood transfusion, advanced cancer stage, and pathological lymphovascular invasion were independent risk factors for cancer recurrence and death in these patients (10). In patients with rectal cancer resection (stage I-III), postoperative EA also did not improve recurrence or mortality with a follow-up duration of 46.1 months when compared to opioids analgesia (11). For patients with stage IV colorectal cancer, one study involving 999 patients showed perioperative EA was not associated with better progression-free or overall survival after surgeries with 17.5 months follow-up (12). In patients underwent laparoscopic colorectal resection for adenocarcinoma, Day et al. showed that postoperative EA had no significant advantage in 5-year overall or disease-free survival than opioids analgesia (13). In contrast, one study revealed that, compared with patient-controlled analgesia, postoperative EA reduces all-cause mortality after open resection of rectal but not colon cancer in patients (14). Furthermore, the results suggested that elder age (>72 years old) and cancer stage (stages 2 and/or 3) were risk factors for death after colon and rectal cancer surgeries. Interestingly, in another study, age was also supposed to be a factor to influence the effects of perioperative EA on oncological outcomes. Gottschalk et al. showed that, although perioperative EA for colorectal cancer surgery did not improve cancer recurrence with a median follow-up time of 1.8 years, a potential benefit was observed in older patients (> 64 years old) (15). Taken together, these findings suggest that the effects of perioperative EA on oncological outcomes after colorectal cancer surgery may be related to the cancer types, stage, patients’ age, and surgery approach, which need further well-designed studies to determine.

Few prospective studies investigated the effects of EA on the cancer recurrence and/or mortality after surgery for colorectal cancer. One multicenter RCT found that, compared with intravenous morphine analgesia, perioperative EA did not improve 5 years disease-free survival in patients underwent colorectal cancer surgery, although perioperative EA significantly reduced postoperative pain during the first 24 h after surgery (16).

However, epidural anesthesia use is also reported beneficial for oncological outcomes in patients after colorectal cancer surgery. One retrospective study revealed that perioperative EA was associated with a better five-year overall survival in patients underwent colorectal cancer surgery. Subgroup analysis also showed that EA contributed to a better overall survival in patients of 80 years and older (17). Another retrospective study also showed that EA improved 5-year survival in patients after colorectal carcinoma surgeries (18).

Christopherson et al. suggested that the potential benefits of perioperative EA depend on cancer staging. They showed that epidural block was associated with enhanced survival in patients without metastases before 1.46 years, but not in patients without metastases after 1.46 years or with metastases (19). Also, Cummings et al. found that perioperative EA improved 5-year survival in patients with nonmetastatic colorectal cancer after open surgery, but did not decrease cancer recurrence (20).

### Effects of Perioperative EA on Gastric Cancer Surgery

For gastric cancer surgeries, the results regarding the effects of EA on oncological outcomes were also conflicting. Most evidences suggest that EA use is not associated with better oncological outcomes in patients underwent gastric cancer surgeries. Compared with intravenous analgesia, perioperative EA was not associated with improved recurrence or survival in patients after gastric cancer surgeries (21, 22). Compared with general anesthesia, epidural anesthesia also showed no effects on the long-term survival of patients after gastric cancer surgeries, but the benefit was observed in younger patients (age up to 64) (23). Furthermore, compared with patient-controlled intravenous analgesia, postoperative EA did not provide better short-term outcomes in patients underwent laparoscopic distal gastrectomy for gastric cancer (99). EA was even associated with a longer length of stay for patients underwent open elective gastrectomies for nonmetastatic cancer (100). Currently, only one retrospective study supported an association between EA use and survival after gastric cancer surgery. The results found that the 5-year overall survival rates were higher in patients receiving general anesthesia combined perioperative EA than that receiving general anesthesia alone (24).

### Effects of Perioperative EA on Esophageal Cancer

Four studies have evaluated the effects of EA on oncological outcomes after esophageal surgeries (25–28). Heinrich et al. showed that postoperative EA did not improve cancer recurrence, 1-year mortality, or 5-year survival after esophagus cancer surgery, although it significantly decreased postoperative opioid consumption and the duration of ICU hospitalization (26). Li et al. also confirmed the benefits of postoperative EA on the short-term outcomes after esophagectomy for cancer, such as attenuating inflammatory response, reducing the incidence of pneumonia and anastomotic leakage, but did not support an association between postoperative EA use and improved 3-year overall recurrence and survival (27). The other two retrospective studies revealed potential benefits of EA on oncological outcomes of EA, of which one study found that perioperative EA was associated with better cancer recurrence and survival after esophageal surgery with 2-year follow-up (28). The other one showed that perioperative EA was associated with better 90-day survival after esophagectomy. Additionally, compared with transthoracic esophagectomy, the five-year survival rates were higher after transhiatal esophagectomy (25), suggesting that the surgical approach may influence the effects of EA on oncological outcomes. Prospective RCTs are needed to assess whether perioperative EA use can improve the cancer recurrence and/or survival after esophageal cancer surgeries.

### Effects of Perioperative EA on Lung Cancer

The current evidences from prospective and retrospective studies do not show a role of perioperative EA use in improving oncological outcomes after lung cancer surgeries. One randomized trial showed that, compared with general anesthesia alone, the combining use with perioperative EA did not improve recurrence-free, overall, or cancer-specific survival in patients after major lung cancer surgery after median follow-up duration of 32 months (29). In patients having non-small-cell lung cancer resection, one retrospective study showed that thoracic epidural analgesia was not associated with better 3-year recurrence-free and overall survival (30). Wu et al. reported that postoperative EA after surgery for non-small cell lung cancer had no association with better 2-year or 5-year recurrence-free survival or overall survival rates. Instead, elder age (≥ 65 years old), male gender, higher body mass index (≥ 25 kg/m^2^), ASA 4, preoperative blood transfusions, pneumonectomy, and postoperative radiation implicated in decreased recurrence-free survival and overall survival (30). Therefore, perioperative EA use appears to not be a factor for oncological outcomes after lung cancer surgeries.

### Effects of Perioperative EA on Ovarian Cancer

The current evidences are conflicting regarding the effects of epidural anesthesia in patients with ovarian cancer surgeries. One study showed that perioperative EA was associated with an increased time to tumor recurrence in patients after ovarian cancer surgery (31). Elias et al. found that the additional use of perioperative EA in general anesthesia was also associated with a lower rate of recurrence in patients with stage III ovarian cancer (32). Tseng et al. reported that perioperative EA was associated with improved progression-free survival (70.8 months follow-up) and overall survival (68.8 months follow-up) in patients with advanced ovarian cancer surgeries (33). However, two studies reported the negative results. One study found that the addition of perioperative EA in general anesthesia did not increase the time to recurrence or overall survival in patients with advanced ovarian cancer surgeries (34). Postoperative EA also did not improve recurrence-free survival and overall survival in patients with advanced-stage ovarian cancer surgery (35).

### Effects of Perioperative EA on Prostate Cancer

The potential impacts of perioperative EA on oncological outcomes in patients with surgeries for prostate cancer are debated. Most evidences point that perioperative EA was not associated with better oncological outcomes in these patients. One study showed that, compared with ketorolac-morphine analgesia, intraoperative and postoperative epidural analgesia did not improve biochemical recurrence-free survival (11.8 years follow-up), 5-year and 10-year cancer-specific survival, or 5 year and 10-year overall survival after open radical prostatectomy (36). Wuethrich et al. also reported that general anesthesia combined with perioperative EA did not improve cancer progression or survival after retropubic radical prostatectomy for prostate cancer with 14 years follow-up (37). One retrospective analysis revealed that intraoperative EA was not, but sufentanil administration was associated with an increased risk of cancer recurrence after retropubic radical prostatectomies with a median follow-up of 38 months (38). Similarly, Tsui et al. demonstrated that, compared with general anesthesia alone, combined general anesthesia with intraoperative EA did not improve disease-free survival following radical prostatectomy for adenocarcinoma with 4.5 years follow-up (39). Currently, only one retrospective study investigated the potential effects of postoperative EA on the long-term outcomes after prostate cancer surgeries, and the results showed that postoperative EA improved cancer recurrence for open prostatectomy surgery (40). Recently, Robot-assisted radical prostatectomy (RARP) has been widely used for prostate cancer and show some potential benefits than open radical prostatectomy, such as improved peri-operative outcomes and functional outcomes (101, 102). Emerging evidence showed that, compared with general anesthesia alone, combined general anesthesia and perioperative EA provided better outcomes in patients undergoing RARP, such as attenuating the severity of postoperative diaphragmatic dysfunction (103) and improving intraoperative ventilation/oxygenation (104). However, no studies have yet investigated the effects of perioperative EA on the oncological outcomes after RARP. It is interesting to determine this in future studies.

### Effects of Perioperative EA on Bladder Cancer

Limited evidence assessed the effects of EA use on the oncological outcomes after bladder cancer surgeries (105). One study evaluated the influence of EA with sufentanil-based epidural analgesia on cancer outcomes in patients receiving radical cystectomy. The results showed that compared with general anesthesia alone, combined general anesthesia with perioperative EA was associated with worse recurrence and disease-free survival for bladder cancer surgeries with 41.4 months follow-up (41), which may be due to the increased opioids use. In another study, Christopher Doiron et al. reported that EA was not associated with cancer-specific survival or overall survival in patients underwent radical cystectomy for bladder cancer (42).

### Effects of Perioperative EA on Hepatocellular Carcinoma

Few studies have evaluated the effects of EA on the oncological outcomes after surgical resection for hepatocellular carcinoma. One retrospective analysis showed no association between perioperative EA use and cancer recurrence or overall survival in patients after surgical resection of hepatocellular carcinoma with a median follow-up time of 64.5 months (43). However, compared with postoperative intravenous analgesia with fentanyl, postoperative EA with morphine increased cancer recurrence and survival but had no effects on recurrence-free survival in patients undergoing resection of hepatocellular carcinoma with a median follow-up time of 4.2 years (44).

Two studies have investigated the association between perioperative EA use and oncological outcomes in patients with colorectal carcinoma liver metastases. Unexpectedly, one study reported that, compared with combined general anesthesia-intraoperative EA, general anesthesia alone may provide a better survival outcome for resected colorectal carcinoma liver metastases with 60 months follow-up (45). However, another study showed that, compared to intravenous analgesia, perioperative EA improved five-year recurrence-free, but not overall survival after colorectal carcinoma liver metastases resection (46).

### Effects of Perioperative EA on Pancreatic Cancer

Epidural analgesia has been used widely in patients underwent pancreatic cancer surgeries due to several advantages such as improved pain control, improved infectious and pulmonary complications (106), although it may be contraindicated in elderly patients for increased risk of epidural-induced hypotension or malfunction (107). Currently, limited evidence put insights on the relationship between perioperative EA use and oncological outcomes after pancreatic cancer surgeries. One study investigated the effects of perioperative EA on oncologic outcomes in patients after resection of pancreatic cancer. The results indicated that perioperative EA was associated with prolonged survival in patients underwent resection of pancreatic adenocarcinoma with a median follow-up time of 437 days (47). Whereas, Alexander et al. reported no association between EA use and recurrence or overall survival in patients underwent radical resection of pancreatic adenocarcinoma, although subgroup analysis revealed a trend towards a longer overall survival associated with perioperative EA in patients with better differentiation of pancreatic adenocarcinoma (48). The concentration of LAs may also influence the effects of EA on the oncological outcomes in patients after pancreatic surgery. One retrospective cohort study reported that, compared with low concentration (0.15%-0.25%) of ropivacaine, intraoperative EA with high concentration (0.375%-0.5%) of ropivacaine was associated with improved overall survival in patients underwent pancreatectomy (108).

## Conclusion

Although it is generally recognized that perioperative EA has advantages in modulating the surgical stress, inflammatory, and immunological responses in patients after cancer surgeries, no definitive evidence yet support or refute an association between the use of perioperative EA and improved cancer recurrence and/or survival. The effects of perioperative EA on oncological outcomes likely depend on the cancer types, cancer staging, patients’ age, opioids use, and the duration of follow-up. Large prospective multicenter RCTs are needed to assess the role of EA in long-term oncological outcomes for cancer surgeries.

## Author Contributions

All authors listed have made a substantial, direct, and intellectual contribution to the work and approved it for publication.

## Funding

This work was supported by Grant No. 81801117 and 81974164 from the National Natural Science Foundation of China; Grant No. 2021M692276 from China Postdoctoral Science Foundation; and Grant No. 21PJ014 from Medical Science and Technology Project of Sichuan Provincial Health Commission.

## Conflict of Interest

The authors declare that the research was conducted in the absence of any commercial or financial relationships that could be construed as a potential conflict of interest.

## Publisher’s Note

All claims expressed in this article are solely those of the authors and do not necessarily represent those of their affiliated organizations, or those of the publisher, the editors and the reviewers. Any product that may be evaluated in this article, or claim that may be made by its manufacturer, is not guaranteed or endorsed by the publisher.
